# Hemisection: A Paradigm Shift in Prolonging Tooth Viability

**DOI:** 10.7759/cureus.62127

**Published:** 2024-06-11

**Authors:** Khyati Manik, Anuja Ikhar, Aditya Patel, Manoj Chandak, Nikhil Mankar, Jay Bhopatkar, Mrudula Shinde

**Affiliations:** 1 Department of Conservative Dentistry and Endodontics, Sharad Pawar Dental College and Hospital, Datta Meghe Institute of Medical Sciences, Wardha, IND; 2 Department of Orthodontics and Dentofacial Orthopaedics, Sharad Pawar Dental College and Hospital, Datta Meghe Institute of Medical Sciences, Wardha, IND

**Keywords:** hemisection, root resection, hemisection, mandibular molar, dental canal, cbct

## Abstract

Splitting a molar means removing or separating the root and the accompanying crown portion. Hemisection of a damaged tooth aids in preserving the tooth structure and the existing alveolar bone surrounding the preserved root while also enabling the installation of a fixed prosthesis. This case report defines hemisection as an effective modality for preserving carious mandibular first molars with periodontal and periapical pathology.

## Introduction

Throughout one's lifetime, the doctor practicing dentistry is expected to care for the patient's tooth function. The loss of back teeth can lead to several unwanted consequences that require preventive and maintenance measures. Choosing the right case can make hemisection a relatively simple, conservative, and affordable treatment with varying odds. In 1964, Simring and Goldberg suggested a correlation between the periodontium and the pulp [1]. Dental canals, apical openings, and lateral and accessory canals are among the communication channels through which the pulp is communicated. Root resection is the process of removing a single root of a tooth at the level of the bifurcation, which allows for removing the infected part and preserving a relatively healthy part of the tooth, preserving its integrity in the socket [2].

Carvalho et al. stated an existence percentage of approximately 93% at 10 years of follow-up in patients who underwent hemisection for molar treatment instead of extraction [3]. The selection of cases and compliance with precise endodontic, surgical, and restorative guidelines are crucial to the success of hemisection. It has been proposed that hemisection ought to be considered before tooth removal as it offers effective, long-standing outcomes [4].

Hemisection is a highly precise procedure. Numerous factors come into play when a clinician selects one treatment plan over another to address a mandibular molar's furcation invasion. These factors include various local considerations such as tooth anatomy, mobility, crown-to-root ratio, attachment loss severity, occlusal relationships, strategic dental value, the patient's general health, and cost factors [5].

Hemisection has been effectively utilized to preserve teeth with furcation involvement. However, it does come with a few drawbacks. Similar to any surgical procedure, it may cause discomfort and unease. Reshaping root surfaces through grinding in the furcation or at the site of hemisection can increase susceptibility to cavities. Often, a positive outcome may be compromised by decay following treatment. Failures of endodontic therapy for any reason can diminish the success of the treatment procedure. Furthermore, when a tooth has lost part of its root support, prosthetic rehabilitation is required to enable independent function or to serve as an abutment for a splint or bridge. The quality of dental restoration plays a crucial role in preventing periodontal damage. Defective margins or improper physiologic form can contribute to destruction. Additionally, an incorrectly shaped occlusal contact area can convert balanced forces into destructive forces, leading to trauma from occlusion and potential failure of hemisection [6].

## Case presentation

**Figure 1:**
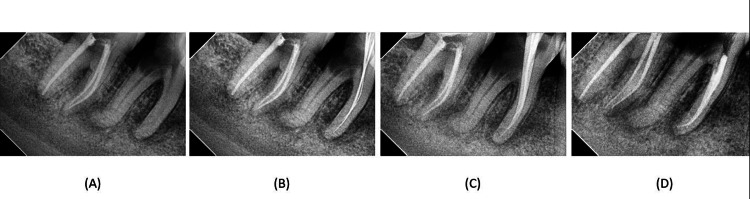
(A) Preoperative radiograph of tooth number 46; (B) working length of tooth number 46; (C) master cone selection of the mesial root of tooth 46; (D) obturation of the mesial root of tooth 46

A 38-year-old female patient living in Wardha contacted the Department of Conservative Dentistry and Endodontics of Sharad Pawar Dental College and Hospital with a complaint of pain in the right upper back of the jaw for a month. When the chief complaint was specified, the pain was spontaneous or persisted for minutes after the stimulus (usually heat, less often cold) was removed. The past medical history, as well as the past dental history of the patient, was non-significant. The X-ray examination revealed radiolucency of the enamel, dentin, and pulp, as well as widening of the periodontal ligament space with the right lower tooth. As a result, a diagnosis of symptomatic irreversible pulpitis with apical periodontitis was made for the right lower first molar (Figure 1A).

The patient received 2% Xylocaine with 1:80,000 adrenaline. Rubber dam isolation was performed. The access cavity was prepared with a Round BR-45 (Mani, Inc., Utsunomiya, Japan) and a safe end Bur EX-24 (Mani, Inc., Utsunomiya, Japan). After removing the pulp tissue from the chamber, four openings were discovered: the mesiobuccal, mesiolingual, and distal openings in typical places, and an additional orifice between the mesiobuccal and mesiolingual was assumed to be a mid-mesial opening. The working length was determined using a Root ZX mini apex locator (JW Morita, Kyoto, Japan) and verified by taking an angled radiograph (mesiobuccal 19 mm, mesiolingual 19.5 mm, midmesial 16 mm) (Figure 1B). Canals were cleaned and shaped using rotary Ni-Ti (Woodpecker 2019, Guilin, China) files up to 20%-4% in the mid-mesial canal and 25%-6% in MB-ML. The canal was irrigated using 3% NaOCl and 0.9% saline, alternatively. Calcium hydroxide (RC Cal, Prime, Palampur, India) and a temporary closed dressing (Neotemp, Neoendo, India) were given, and the patient was further recalled after seven days.

On the second appointment, the patient was completely asymptomatic. The temporary dressing was removed, and all the canals were sonically activated with 17% EDTA to facilitate calcium hydroxide removal. All the canals were then irrigated using 3% NaOCl and 0.9% saline, alternatively. The Gutta-percha master cones were selected (Figure 1C). Obturation was carried out with master cones and epoxy resin-based sealer (Diaproseal, DiaDent, Burnaby, Canada) (Figure 1D).

**Figure 2:**
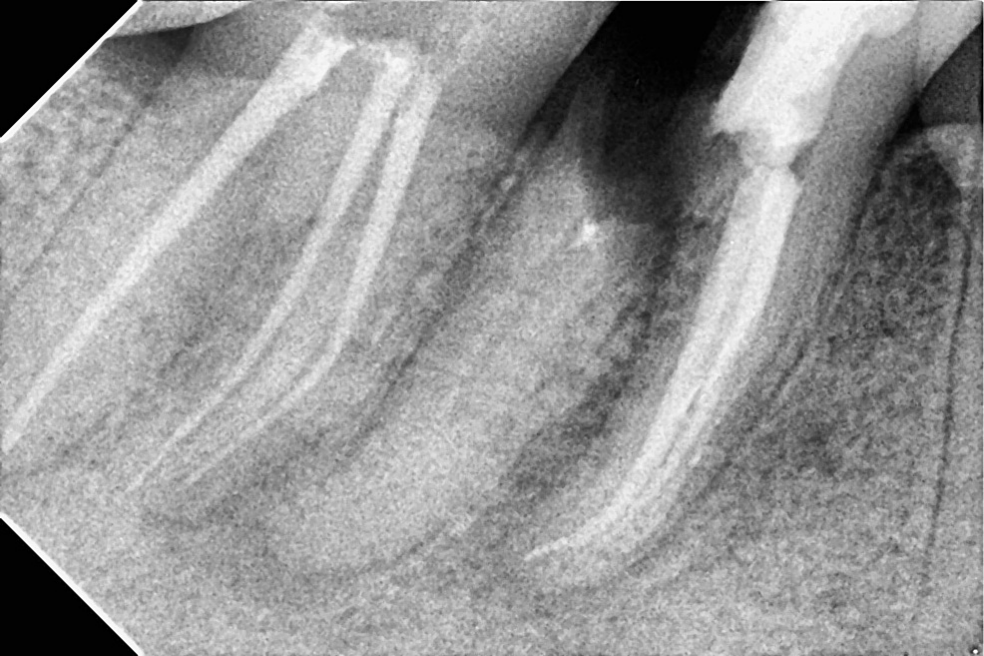
Postoperative endodontic restoration of tooth number 46

A composite restoration (Spectrum, Dentsply, Charlotte, North Carolina) was performed after endodontic treatment (Figure 2).

**Figure 3:**
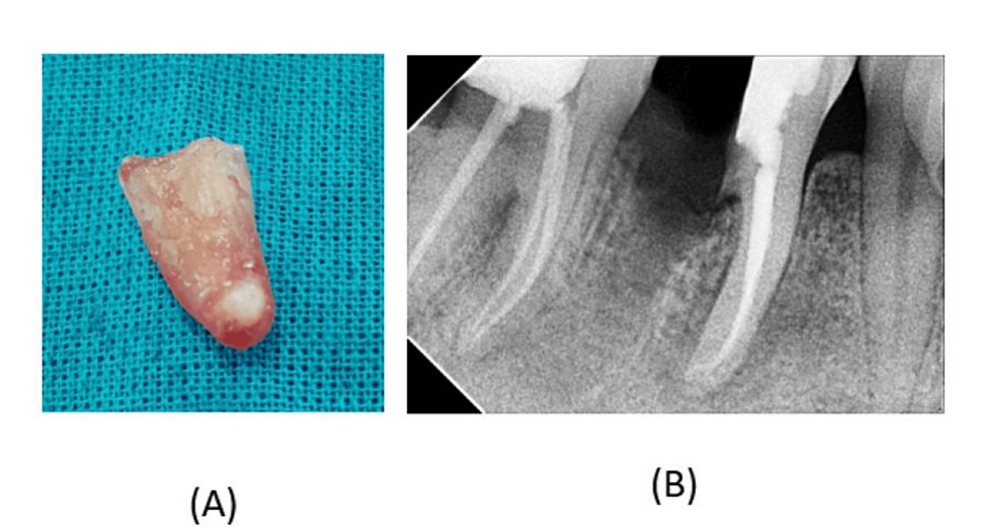
(A) Hemisection of the distal root of tooth number 46; (B) postoperative radiograph of tooth 46

Following the endodontic procedure, hemisection of the distal root was undertaken. This case was indicated for hemisection because of the carious extension into the furcal area of tooth number 46. The procedure was performed under local anesthesia. The tooth was cut vertically through the “buccal and lingual developmental grooves,” passing from the chamber of the pulp and bifurcation to remove the distal portion of the root and crown. The distal portion of the root was removed without causing any traumatic injury using a high-speed, long, narrow burr toward the bifurcation area (Figure 3).

**Figure 4:**
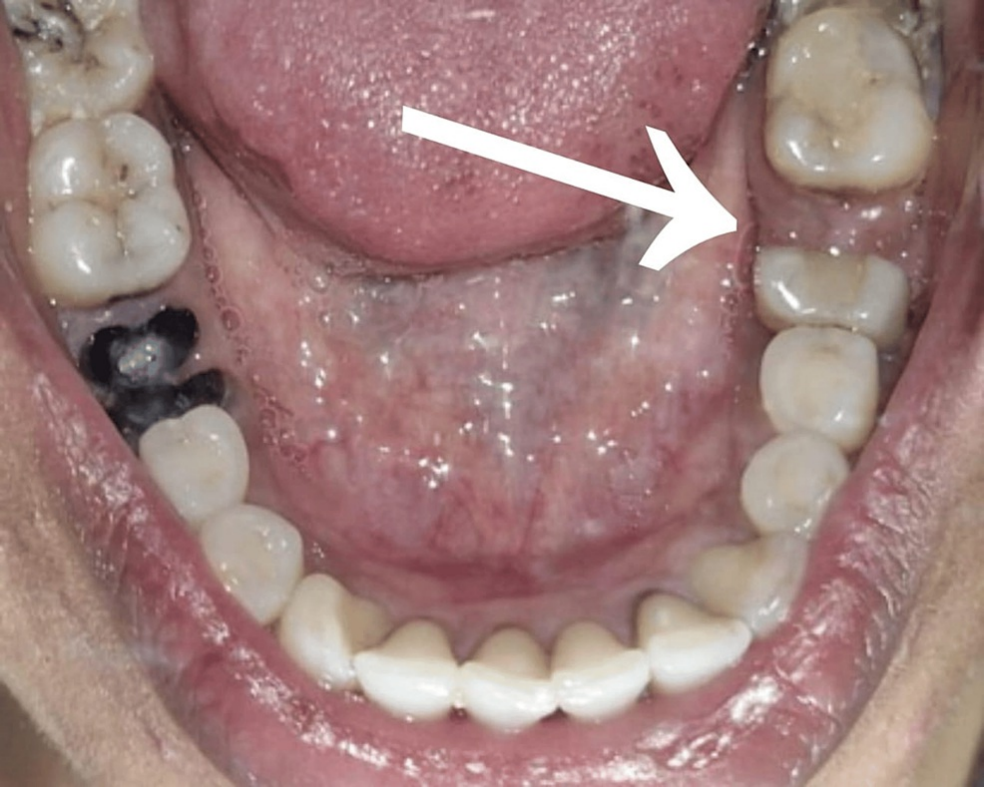
Postoperative clinical photograph of tooth number 46

**Figure 5:**
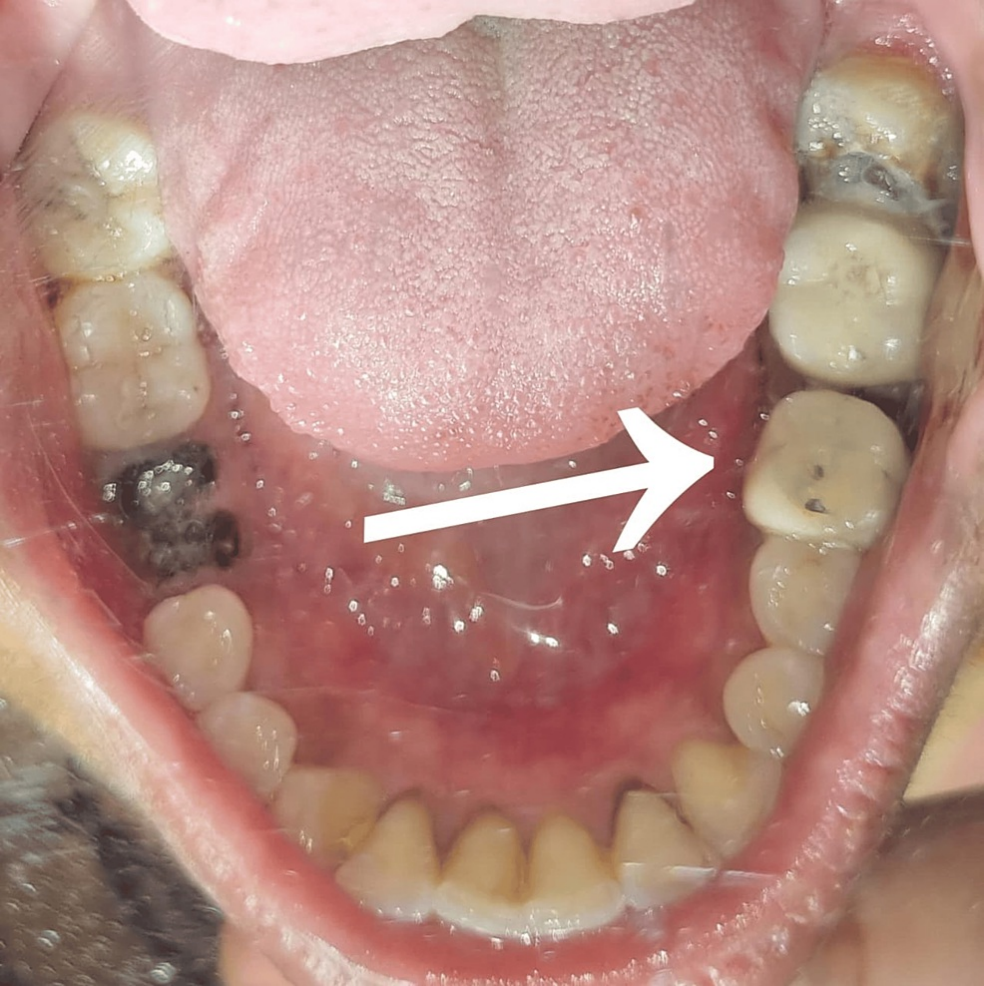
Occlusal view of the porcelain-fused-to-metal prosthesis of tooth number 46

**Figure 6:**
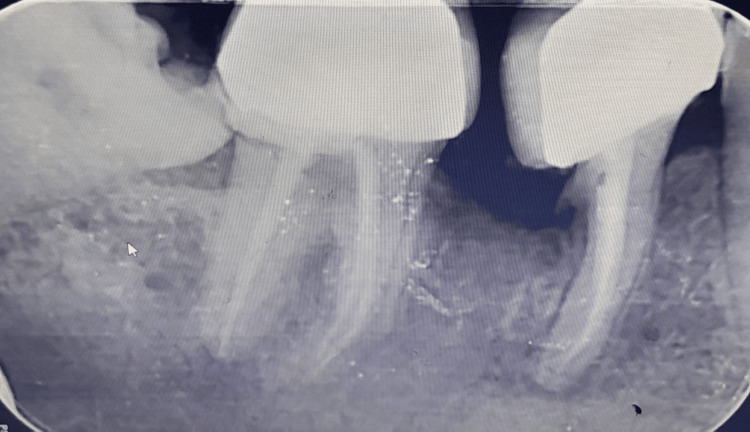
Follow-up radiograph after six months

Figure 4 displays a postoperative clinical photograph. Tooth preparation of the mesial portion of the lower right first molar and second molar was performed, followed by a porcelain-fused-to-metal prosthesis (Figure 5). Following a hemisection of the distal root of the right mandibular first molar, several normal periodontal changes were observed over a six-month follow-up period. Initially, there may be transient inflammation and discomfort as the periodontal tissues respond to the surgical intervention. Over time, this inflammation subsided, and the periodontal health was stabilized. The alveolar bone around the remaining root underwent remodeling, resulting in changes in both bone density and contour. Periodontal probing depths around the remaining root were decreased, reflecting improved periodontal attachment and health. Furthermore, the gingival margin reestablished itself in a manner that maintains periodontal health, provided that good oral hygiene is maintained. The radiographic findings revealed a progressive formation of the alveolar bone around the area of the resected root, with no evidence of bone loss or pathological alterations. The periodontal ligament space appears normal, reflecting healthy adaptation of the remaining root. The lamina dura appeared normal. Furthermore, there was resolution of radiolucency around the mesial root of tooth number 46 (Figure 6).

## Discussion

The success rate of therapy is consistent with comprehensive clinical information, diagnosis and prognosis, and a multidisciplinary treatment plan. The damage can start as an individual, either through the periodontium or the pulp, but at the moment of onset, a combined effect is possible. Exclusively employing endodontic treatment in this situation will result in the healing of the periapical lesion exclusively at its origin. Periodontal treatment alone can only result in the healing of the crestal bone. The treated lesion has not fully healed, as it remains irritated and inflamed due to the lack of proper treatment in the untreated section [7].

Furcal involvement in endo-perio lesions is a primary challenge for the treatment plan. There are various treatments for furcation symptoms, such as open flap removal, bone resection, regenerative procedures, and root section [7]. Langer et al. found that endodontic or restorative problems, such as a root fracture and untreated lesions and caries, were found to be responsible for 36% of root-extracted teeth failing within 10 years. This was more common than periodontal problems. Therefore, the practitioner should try to conserve as much tooth structure as feasible [8].

Basten et al. stated that 92% of removed teeth had a median survival time, but most failures were due to caries or endodontic and strategic factors [9]. In cases of progressive bone loss with bifurcation, hemisection was employed, which implied the splitting of the molar and the surgical removal of both the diseased root and crown [10]. The chief benefit of this action is that the bifurcation is accompanied by a tooth with multiple roots and a fragile single-rooted tooth, creating a fortunate environment for maintaining oral hygiene [11].

In a comprehensive study, Park et al. conducted root resection therapy on 691 molars in 579 patients [12]. The analysis of factors from 342 of 402 molars followed up for over a year revealed that root resection for periodontal issues yielded a more favorable prognosis than for non-periodontal concerns. The study highlighted the importance of the remaining roots having over 50% bone support for achieving positive outcomes. This crucial finding has the potential to significantly enhance the predictability of root resection therapy [12].

Bühler's research in 1988 revealed a 32% failure rate in hemisection cases due to endodontic pathology and root fracture. However, long-term studies have demonstrated greater success in this area [13].

Shafiq successfully performed a procedure in which he removed the mesial root and inserted a three-unit bridge connecting the remaining root to the adjacent second premolar [14]. This innovative approach has kept the bridge in place for over a year. Shafiq suggests that retaining a portion of the tooth can significantly extend the lifespan of a prosthesis, providing patients with the option of opting for tooth hemisection or root amputation instead of extraction [14].

## Conclusions

The hemisection of the distal root of the mandibular first molar is a complex procedure aimed at preserving part of the tooth while removing the problematic root. Following the surgical procedure, the follow-up period is important and typically spans several months, with regular checkups to monitor healing and ensure the success of the treatment. A fixed prosthesis, often a dental crown or bridge, is placed to restore functionality and esthetics. Special consideration is given to the occlusal load on the remaining root to prevent overloading and potential failure. This involves careful adjustment of the bite, ensuring proper distribution of forces, and possibly incorporating occlusal guards to protect the tooth during healing and function. Regular monitoring and adjustments are essential to maintain the health and functionality of the treated tooth. Conservative treatment of carious teeth with multiple roots in adults saves dentition and lessens the financial load, mental suffering, and occlusion disorders associated with tooth loss. Subsequently, consistent periodontic care and proper coronal restoration of the extracted teeth are essential for their long-standing existence.
